# Ecological Observations of Native *Geocoris pallens* and *G. punctipes* Populations in the Great Basin Desert of Southwestern Utah

**DOI:** 10.1155/2013/465108

**Published:** 2013

**Authors:** Meredith C. Schuman, Danny Kessler, Ian T. Baldwin

**Affiliations:** Department of Molecular Ecology, Max Planck Institute for Chemical Ecology, Hans-Knöll-Straße 8, 07745 Jena, Germany

## Abstract

Big-eyed bugs (*Geocoris* spp. Fallén, Hemiptera: Lygaeidae) are ubiquitous, omnivorous insect predators whose plant feeding behavior raises the question of whether they benefit or harm plants. However, several studies have investigated both the potential of *Geocoris* spp. to serve as biological control agents in agriculture and their importance as agents of plant indirect defense in nature. These studies have demonstrated that *Geocoris* spp. effectively reduce herbivore populations and increase plant yield. Previous work has also indicated that *Geocoris* spp. respond to visual and olfactory cues when foraging and choosing their prey and that associative learning of prey and plant cues informs their foraging strategies. For these reasons, *Geocoris* spp. have become models for the study of tritrophic plant-herbivore-predator interactions. Here, we present detailed images and ecological observations of *G. pallens* Stål and *G. punctipes* (Say) native to the Great Basin Desert of southwestern Utah, including observations of their life histories and color morphs, dynamics of their predatory feeding behavior and prey choice over space and time, and novel aspects of *Geocoris* spp.’s relationships to their host plants. These observations open up new areas to be explored regarding the behavior of *Geocoris* spp. and their interactions with plant and herbivore populations.

## 1. Introduction

*Geocoris* spp. Fallén (Hemiptera: Lygaeidae), commonly known as big-eyed bugs, are generalist insect omnivores which occur naturally worldwide [1]. *Geocoris* spp. are well known to prey on a variety of insects, including several economically important agricultural pests [1, 2] but have also been reported to feed on plant material [1–6], particularly seeds [1, 5, 7]. Several studies in laboratories [1, 4, 6, 8–14], agricultural fields [1, 8, 15–21], and natural habitats [22–31] have investigated the potential of multiple *Geocoris* spp.—including *G. bullatus* (Say) [1], *G. ochropterus* (Fieber) [10], *G. pallens* Stål [1, 20–22, 24, 26–31], *G. proteus* Distant [32], *G. punctipes* (Say) [4, 6, 9, 11–18, 20, 21, 33], *G. uliginosus* (Say) [16, 19, 33], and *G. varius* (Uhler) [32]—to serve as biological control agents to protect plants against herbivores. These studies have found that individual *Geocoris* spp. accept a variety of insect prey, and the field studies have also shown that *Geocoris* spp. reduce herbivore populations [1, 15, 17, 18, 20–24, 26–29] (but see [25]) and increase plant yield [23, 31]. Thus, despite plant feeding, the net effect of *Geocoris* spp.-plant interactions is usually beneficial to plants [34], and *Geocoris* spp. can be effective biological control agents in many agricultural systems. The most important consequence of plant feeding by *Geocoris* spp. may be that it renders them more directly susceptible to agricultural pesticides [6].

*Geocoris* spp. adults lay their eggs on plants in nature, or on moist cotton or paper cellulose in the laboratory. Life history traits have been characterized in laboratory colonies of *G. atricolor* Montandon [35], *G. bullatus* [1], *G. lubra* Kirkaldy [36], *G. pallens* [1, 35], and *G. punctipes* [35, 37]. The speed of development from egg to adult correlates positively with temperature between 21°C and 37°C; outside this range, eggs are not viable [1, 35, 36]. The photoperiod associated with the most rapid development differs among species; the photoperiod for which development is slowest may correspond to the diapause-inducing photoperiod for a species [36]. Eggs hatch after ca. 1–3 weeks depending on temperature (higher temperature = faster development) [1, 36, 37], and nymphs develop through five stages over ca. 1 month before reaching adulthood [36, 37]; nymph viability was found to be higher at 27°C than at 24°C for *G. lubra*, but higher at 24°C than at 27°C for *G. punctipes* [35, 36]. Adults can survive from 1 week to nearly 4 months in captivity [37].

*Geocoris* spp. feed on a combination of insect prey and plant material [1, 2, 4, 5, 7]. They can survive if given a water source and either insect prey or plant seeds, but diets combining insects with seeds or seed pods decrease development time and increase survival rates and fecundity; *Geocoris* spp. may even require seeds or seed pods in order to complete development [1, 5, 38]. This may be due in part to the fact that *Geocoris* spp. prey on many different insects of varying nutritional value. Interestingly, although *Helicoverpa zea* Boddie (Lepidoptera: Noctuidae) eggs are higher quality food for *G. punctipes* than are *Acyrthosiphon pisum* Harris (Hemiptera: Aphididae), *G. punctipes* more often preyed on *A. pisum* in choice tests [5, 38]. Seed pods and seeds are thus important nutritional resources for *Geocoris* spp. [1, 2, 5, 7, 38]. However, because leaf feeding has not been shown to increase survival in comparison to a water-only diet, it is thought that leaves serve only as a water source [3, 4].

We study the ecological interactions of the wild tobacco *Nicotiana attenuata* Torr. ex S. Watson (Solanales: Solanaceae) in its native habitat, the Great Basin Desert of the southwestern USA. The postfire germination behavior of *N. attenuata* creates large monocultures of plants that host a diverse insect herbivore community [39]. This herbivore community includes several specialists on Solanaceae: *Corimelaena extensa* Uhler (Hemiptera: Thyreocoridae) [39], *Epitrix hirtipennis* (Melsheimer) and *E. subcrinita* LeConte (Coleoptera: Chrysomelidae) [28], *Manduca quinquemaculata* (Haworth) and *M. sexta* (Linnaeus) (Lepidoptera: Sphingidae), and *Tupiocoris notatus* (Distant) (Hemiptera: Miridae) [23]; the generalist herbivores *Spodoptera* spp. Guenée (Lepidoptera: Noctuidae) and *Trimerotropis* spp. (Orthoptera: Acrididae) [40]; and opportunistic herbivores which attack only poorly-defended plants, such as *Empoasca* spp. Walsh (Hemiptera: Cicadellidae) [41, 42] and *Heliothis* spp. Ochsenheimer (Lepidoptera: Noctuidae) [43]. *G. pallens* is a common predator of herbivores on *N. attenuata*, and both *G. pallens* and *G. punctipes* can be found on *N. attenuata* or neighboring plant species during *N. attenuata*’s growing season [22, 31]. *Geocoris* spp. respond to volatiles emitted from *N. attenuata* after herbivory by removing more herbivores from emitting plants [22, 26, 27, 29–31], resulting in a fitness benefit for plants [31].

Here, we present quantitative and qualitative observations and high-resolution images of morphology and behavior for *G. pallens* and *G. punctipes* co-occurring with *N. attenuata*. We have observed aspects of the life history, host plant, and insect prey preferences of both species. For *G. pallens*, we have also made detailed recordings of feeding behavior with a high-resolution macro lens (courtesy of A. Shillabeer with Merit Motion Pictures); quantified variation in predation activity of subpopulations with respect to the lepidopteran herbivore *M. sexta*; assayed the inclination of different generations of nymphs and adults from a single wild population to feed on *M. sexta* over a season; recorded increased occurrence of *Geocoris pallens* on wilting *N. attenuata* plants; and demonstrated that *G. pallens* can, in fact, survive when provided only with water and vegetative plant tissue.

## 2. Methods

### 2.1. Study Sites and Insect Collections

*Geocoris pallens* and *G. punctipes* were assayed in and collected from Lytle Preserve in the Great Basin Desert of southwestern Utah, USA (latitude 37.146, longitude −114.020), where we have annual field plantations of *N. attenuata*, and from a nearby location where a native *N. attenuata* population could be found from 2007 to 2009 after a 2006 burn (latitude 37.077, longitude −113.833). In May and June 2009, we collected adults and nymphs of *G. pallens* from the native *N. attenuata* population (four collections of 73 insects (19% adults), 31 insects (71%), 99 insects (58%), and 107 insects (95%)), allowed adults to mate and lay eggs, and observed the eggs through development to adults. These collections, together with ca. 100 insects collected from Lytle and a nearby wash, were used to start a colony of *G. pallens* at our institute in Jena, Germany. This colony has since received annual inputs from field collections. *G. punctipes* adults were also collected in June 2009 and used to start a colony in Jena. The colonies are fed a diet of Nutrimac (sterilized *Ephestia kuehniella* eggs, Biobest N. V.), *Manduca sexta* and *Spodoptera littoralis* (Boisduval) eggs and larvae, and *N. attenuata* green tissue and seeds, with additional water provided using moist dental rolls in microcentrifuge tubes containing tap water. They are kept in 9 L food-quality plastic boxes (Lock & Lock) with two holes in each lid of ca. 8 cm diameter each covered with a fine mesh, and containing paper towels to provide structure for oviposition and hiding places, inside a growth chamber (Snijders Scientific, http://www.snijders-scientific.nl/cooling-and-freezing-systems/) with 16 h D/8 h N (06:00–22:00 D/22:00–06:00 N), 26/22°C, daylight provided by Osram L 36 W/77 fluorescent lamps (http://www.osram.com/) at 50% power, 65% RH, and ventilation by PAPST type 4656 N fans (http://www.ebmpapst.com/en/).

### 2.2. Images of Geocoris pallens and G. punctipes

**Figure 1:**
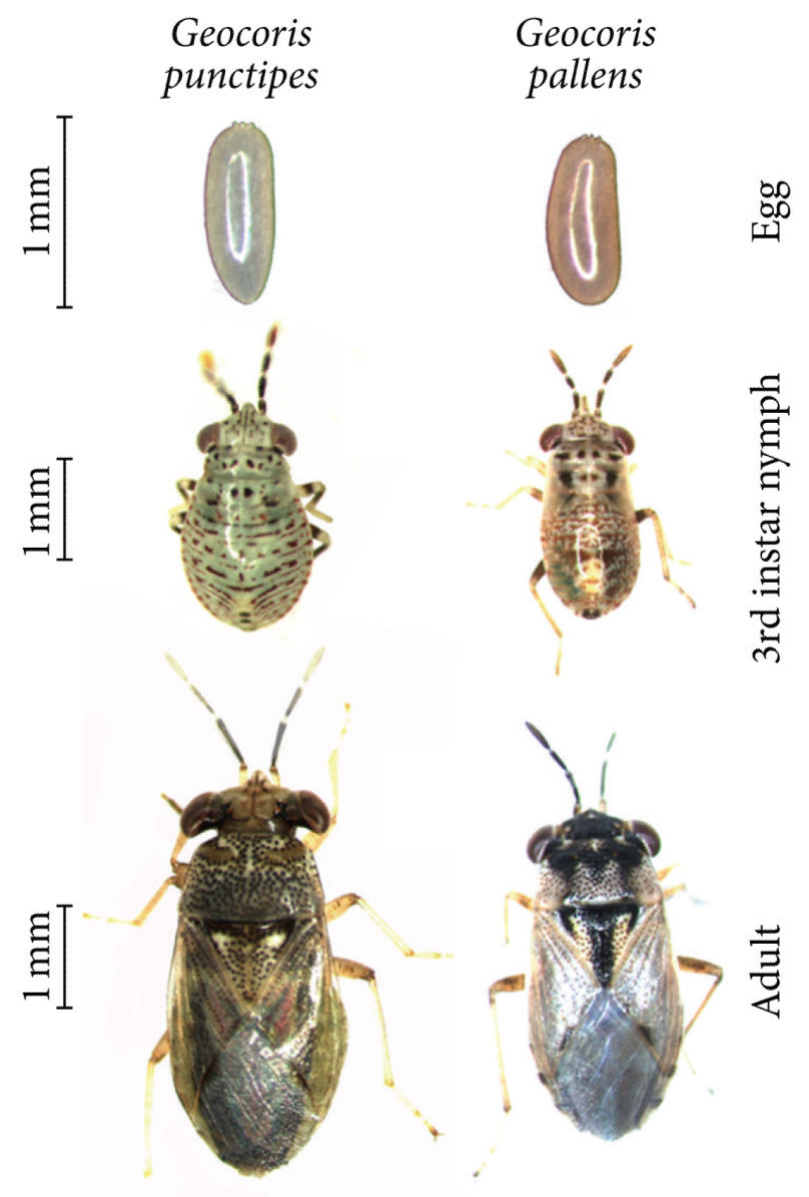
Comparison between *Geocoris punctipes* and *G. pallens* collected from the Great Basin Desert in southwestern Utah.

**Figure 2:**
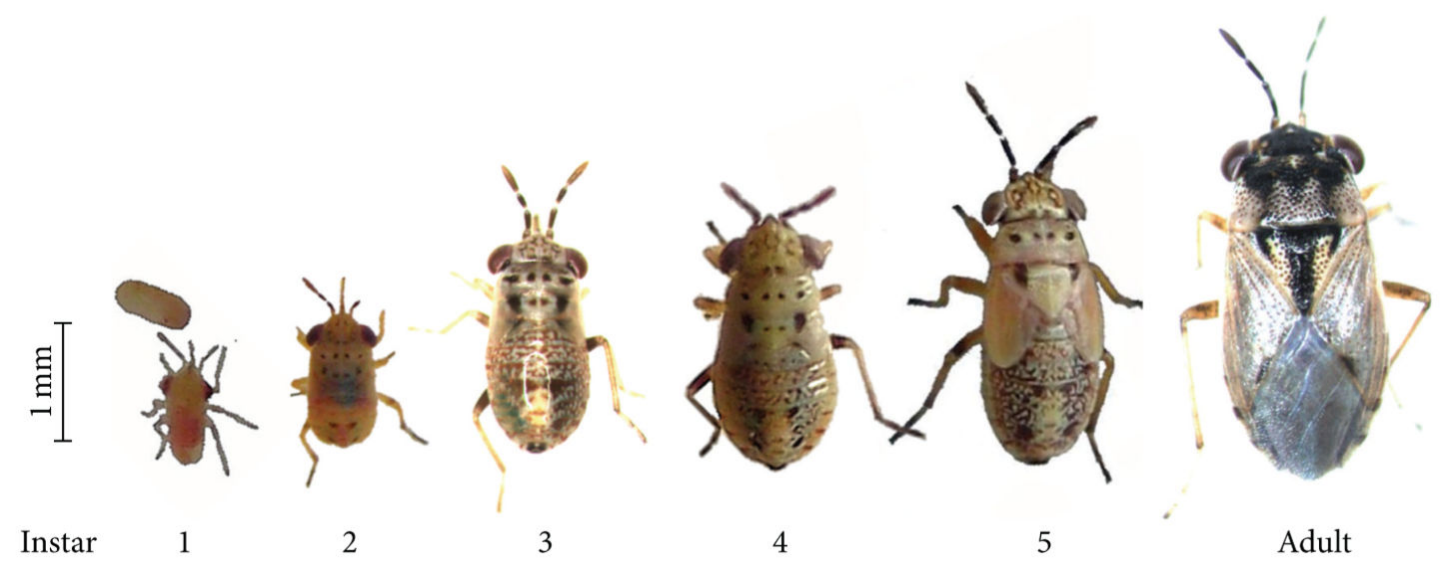
Larval and adult stages of *G. pallens* from the Great Basin Desert in southwestern Utah. *Geocoris* spp. have five nymphal instars.

**Figure 3:**
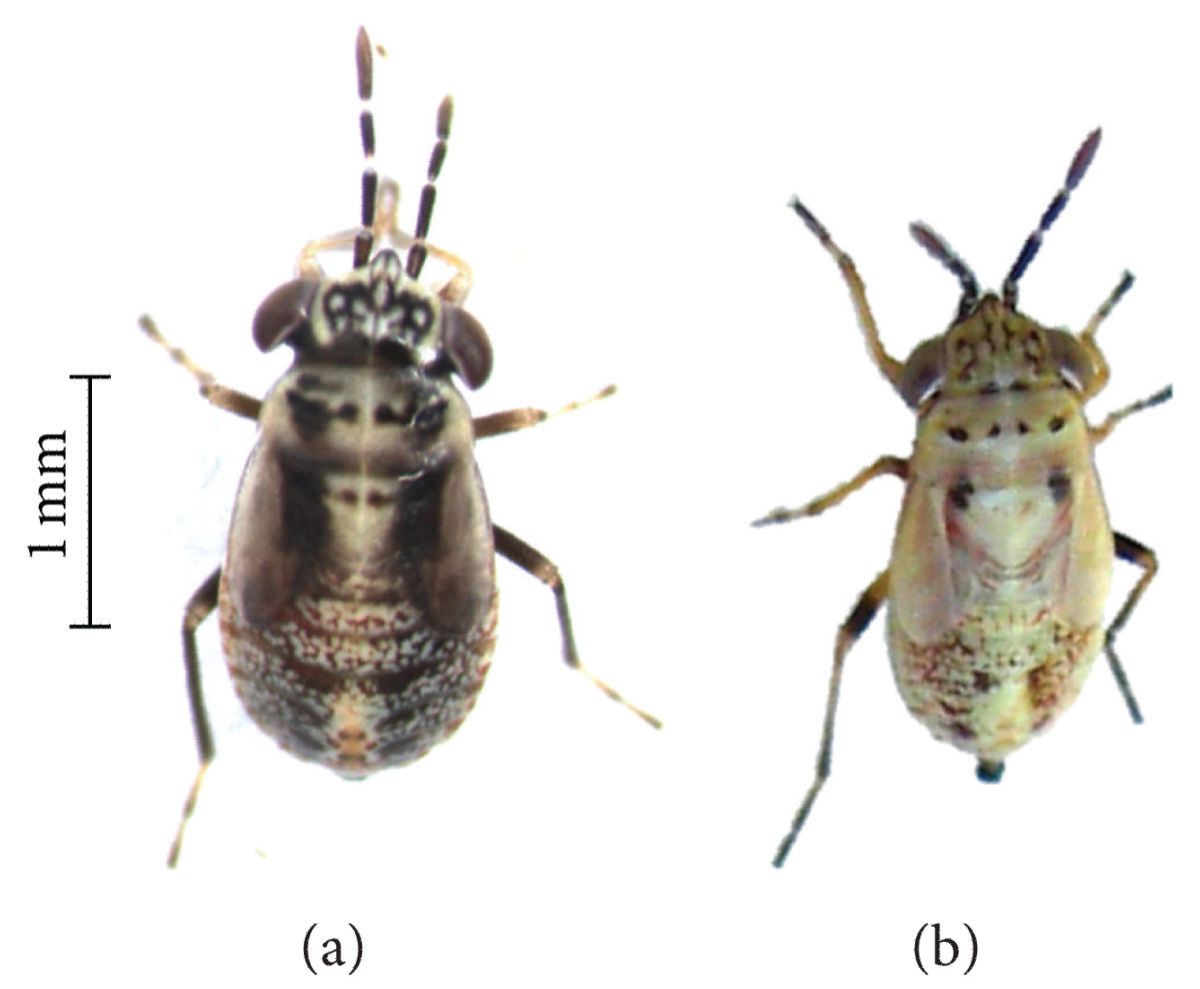
Color morphs of *G. pallens* nymphs. The dark (a) and the more common light (b) color morph are shown in the fourth instar. Size differences are not characteristic of the morphs but are rather due to individual differences.

**Figure 4:**
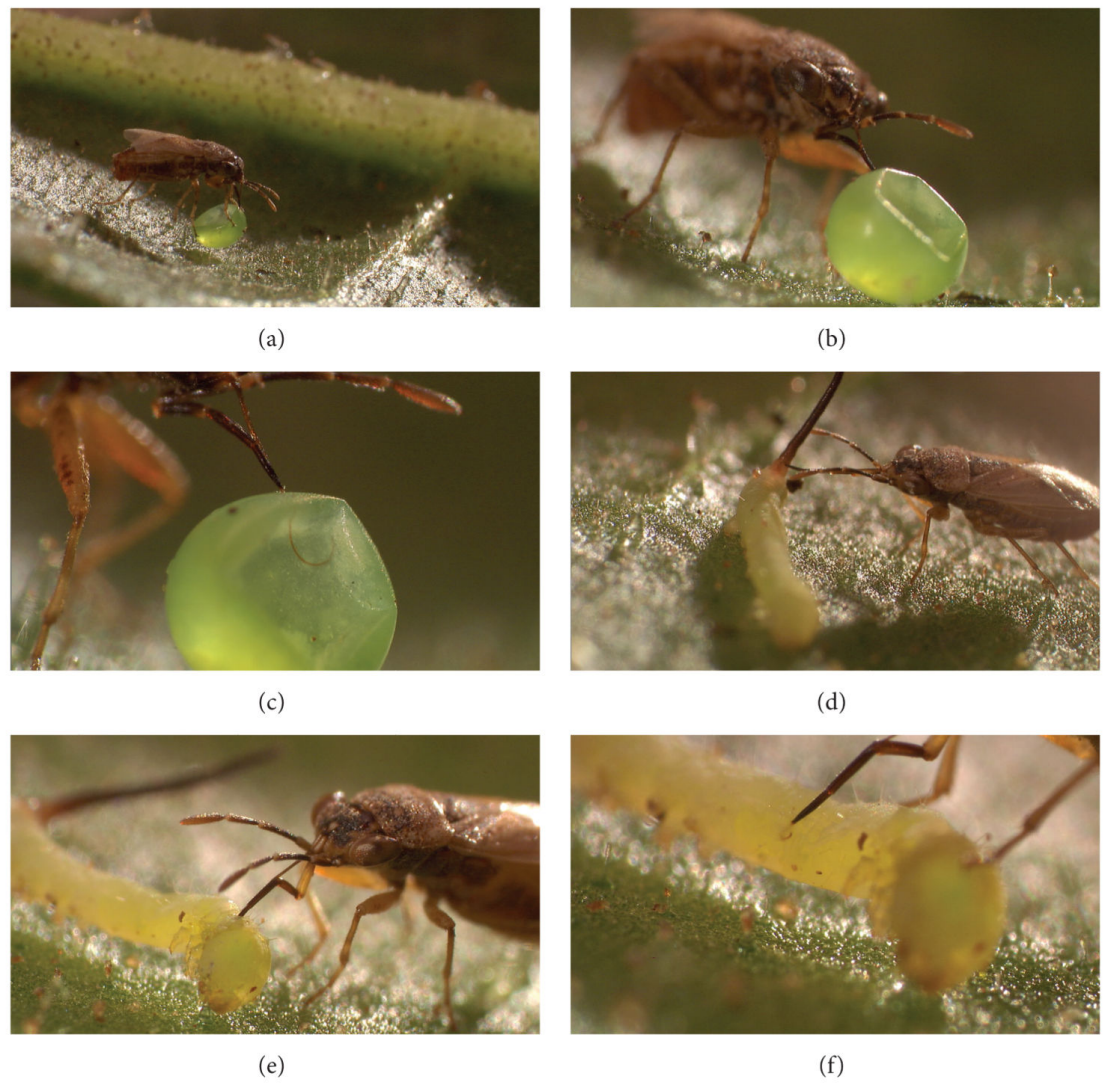
*G. pallens* feeding on a *Manduca sexta* egg (a)–(c) or larva (d)–(f). Note the flexible stylet clearly visible inside the egg in (c). Copyright: Merit Motion Pictures, Winnipeg, MB, Canada.

Pictures were taken of insects collected from the native *N. attenuata* population in 2009 (Figures 1, 2, and 3) or from Lytle in 2011 (Figure 4). Images in Figures 1–3 are from an Axiocam HRc connected to a stereomicroscope SV 11 and captured with AxioVision 4.0 software (Zeiss, http://www.zeiss.com/corporate/en_de/home.html; Figure 1, Figure 2 instar 3 and adult), or from a Powershot SD1000 camera (Canon, Inc., http://www.usa.canon.com/cusa/home; Figure 2 instars 1, 2, 4, and 5; Figure 3). Images in Figure 4 were taken with a probe lens (Innovision Optics, http://www.innovision-optics.com/) by A. Shillabeer and kindly provided by Merit Motion Pictures (Winnipeg, MB, Canada). The probe lens permits the capture of HD macro images with an unusually large depth of field.

### 2.3. Egg Predation Assays

**Figure 5:**
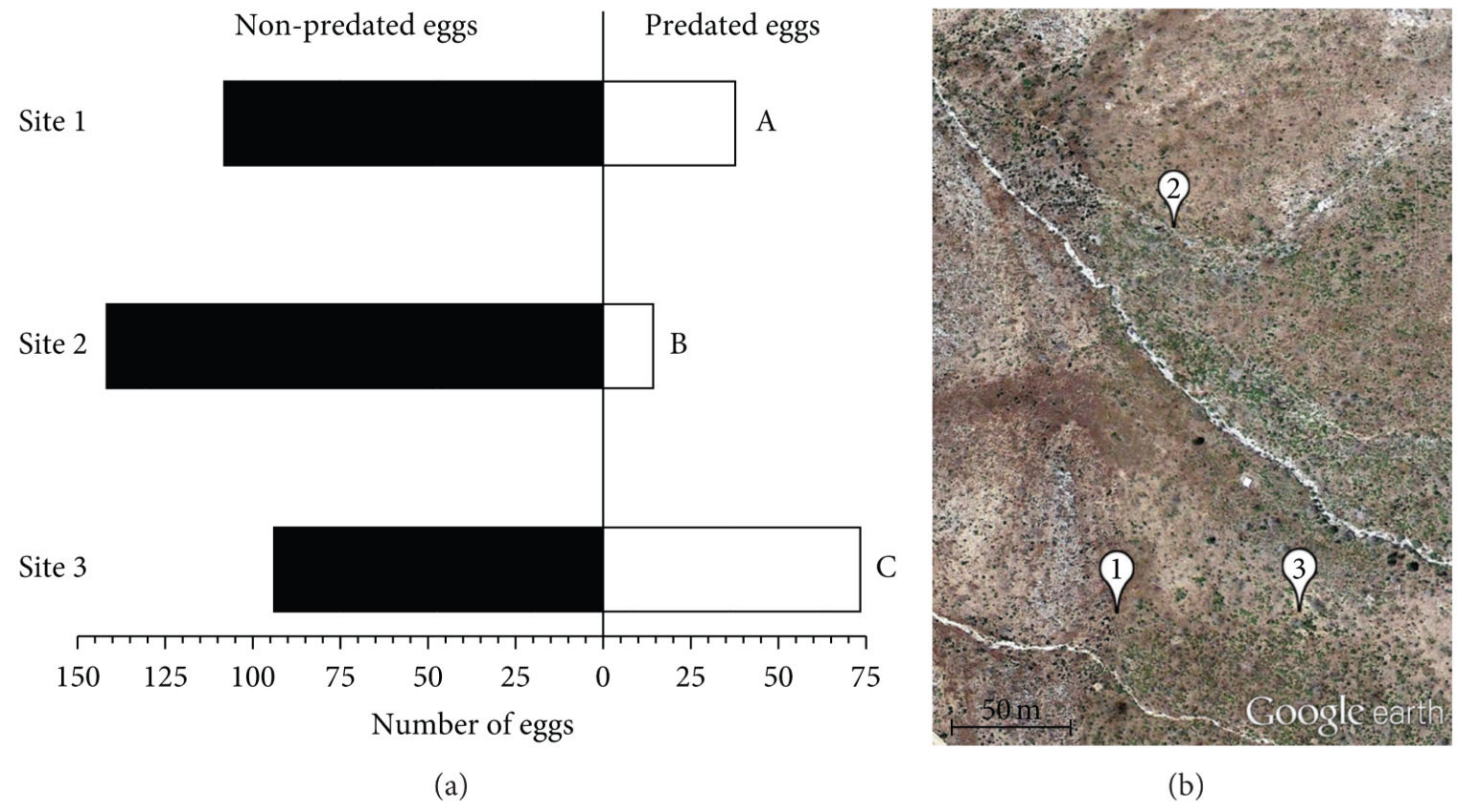
*Geocoris* spp. predation activity differs among sites within a native *N. attenuata* population. (a) The graph shows numbers of *M. sexta* eggs predated over 36 h by *Geocoris* spp. Letters indicate significant differences between sites in Bonferroni-corrected pairwise Fisher’s exact tests, *P* < 0.003. (b) Sites were clusters of plants ca. 50–100 m apart.

Although *M. quinquemaculata* and *M. sexta* moths oviposit in native *N. attenuata* populations, the number of eggs is usually not sufficient for experiments except in outbreak years. Thus, we use *M. sexta* eggs and larvae from lab colonies for many field assays. In June 2007, *M. sexta* eggs purchased from North Carolina State University were frozen to kill developing larvae and thus prevent hatching, then thawed and used to assay native *Geocoris* spp. predation activity in a wild population of *Nicotiana attenuata* growing on a recent burn (see Section 2.1). Five eggs were glued with an *α*-cellulose glue (KVS, Leuna, Germany)—which does not damage plants, induce volatile emission, or prevent egg predation—to the underside of a similarly sized, intact lower stem leaf in a standardized position (as in [22]) on 35 plants per location, in three locations within the native *N. attenuata* population (Figure 5). After 36 h, empty eggs with intact shells containing visible puncture holes typical of *Geocoris* feeding (Figure 4(c)) were counted as predated, and intact eggs were counted as non-predated. Missing eggs were not included in counts.

### 2.4. “Feeder/Non-Feeder” Assays

**Figure 6:**
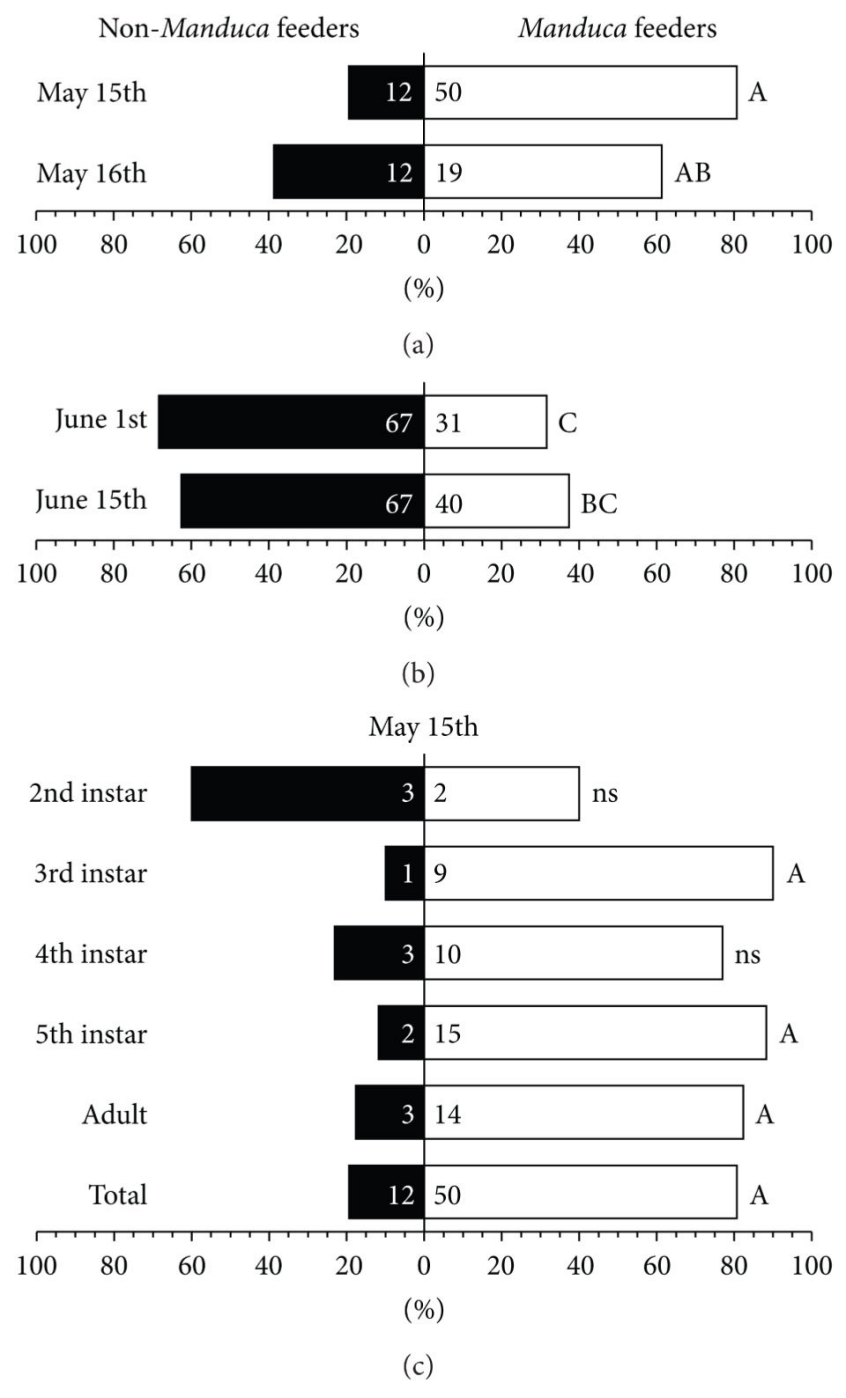
*G. pallens* collected from a single wild *N. attenuata* population vary in their tendency to eat *M. sexta* eggs, but different stages in a single collection do not. Graphs show percentages of *G. pallens* in collections which ate *M. sexta* eggs within 72 h in no-choice assays; counts are in bars. Letters indicate significant differences in Bonferroni-corrected pairwise Fisher’s exact tests across all groups, *P* < 0.05; ns: no significant difference to any other group. (a) Individuals collected in May and tested immediately after collection show a similar tendency to eat *M. sexta* eggs (61–81%). (b) Individuals collected at two dates in June and tested 24–48 h later, after transportation to a laboratory and a short adjustment period, also show a similar tendency to eat *M. sexta* eggs (32–37%), although the tendency is lower than for the May collections. This could be due either to a shift in the population’s tendency to eat *M. sexta* eggs or to transportation and changed environmental conditions. (c) The May 15th population had a fairly even distribution of different nymphal stages and adults, which did not differ significantly in their tendency to eat *M. sexta* eggs.

We observed in many years that *G. pallens* prey on small bugs from invasive stork’s bill ground cover plants (*Erodium cicutarium* (L.) L’Hér. ex Aiton, Geraniales: Geraniaceae) in April and May and move to *N. attenuata* plants later in the spring, as *E. cicutarium* plants are drying up. On *N. attenuata*, *G. pallens* prey on flea beetles (*Epitrix* spp.), which are usually the first herbivores on *N. attenuata*, and mirids (*T. notatus*) which arrive on *N. attenuata* as plants begin to elongate. If *Manduca* spp. moths oviposit on *N. attenuata* (which they often do when pollinating flowers), *G. pallens* will begin to eat *Manduca* spp. eggs and young larvae [23, 30]. We conducted feeding assays to quantify the tendency of two generations of *G. pallens* nymphs and adults to feed on *M. sexta* eggs (Figure 6). On four separate days in May and June 2009, *G. pallens* were collected from a native *N. attenuata* population (see Section 2.1); Table 1 shows the distribution of adults and nymphs in each collection. May collections were tested at the field station immediately after collection, and June collections were tested after transportation to the laboratory in Germany, within 48 h after collection (during which *G. pallens* individuals had access to a variety of field-collected plant and insect food and could adapt to the new conditions). Each individual was put with a piece of damp cotton and a single *M. sexta* egg into a 30 mL Dixie plastic cup (http://www.dixie.com/) with a lid containing small air holes and left for 72 h; cups were kept by a window in a shaded travel trailer at the field station (May assays) or in a laboratory (June assays), and water from an underground spring (May assays) or from a tap (June assays) was added to the cotton daily. *G. pallens* individuals which had eaten the egg within 72 h were counted as feeders, and those which had not were counted as nonfeeders. From the June 15th collection, two of the *M. sexta* eggs hatched and the larvae were eaten; these *G. pallens* were also counted as feeders. Native *Manduca* spp. oviposition in the field at this time was not sufficient for feeding assays, and the *M. sexta* for the May assays in 2009 were kindly provided by C. Miles of the State University of New York at Binghamton; the *M. sexta* for June assays came from an in-house colony of the same original stock as the Binghamton colony.

### 2.5. G. pallens Populations around Wilting versus Healthy Plants

**Figure 7:**
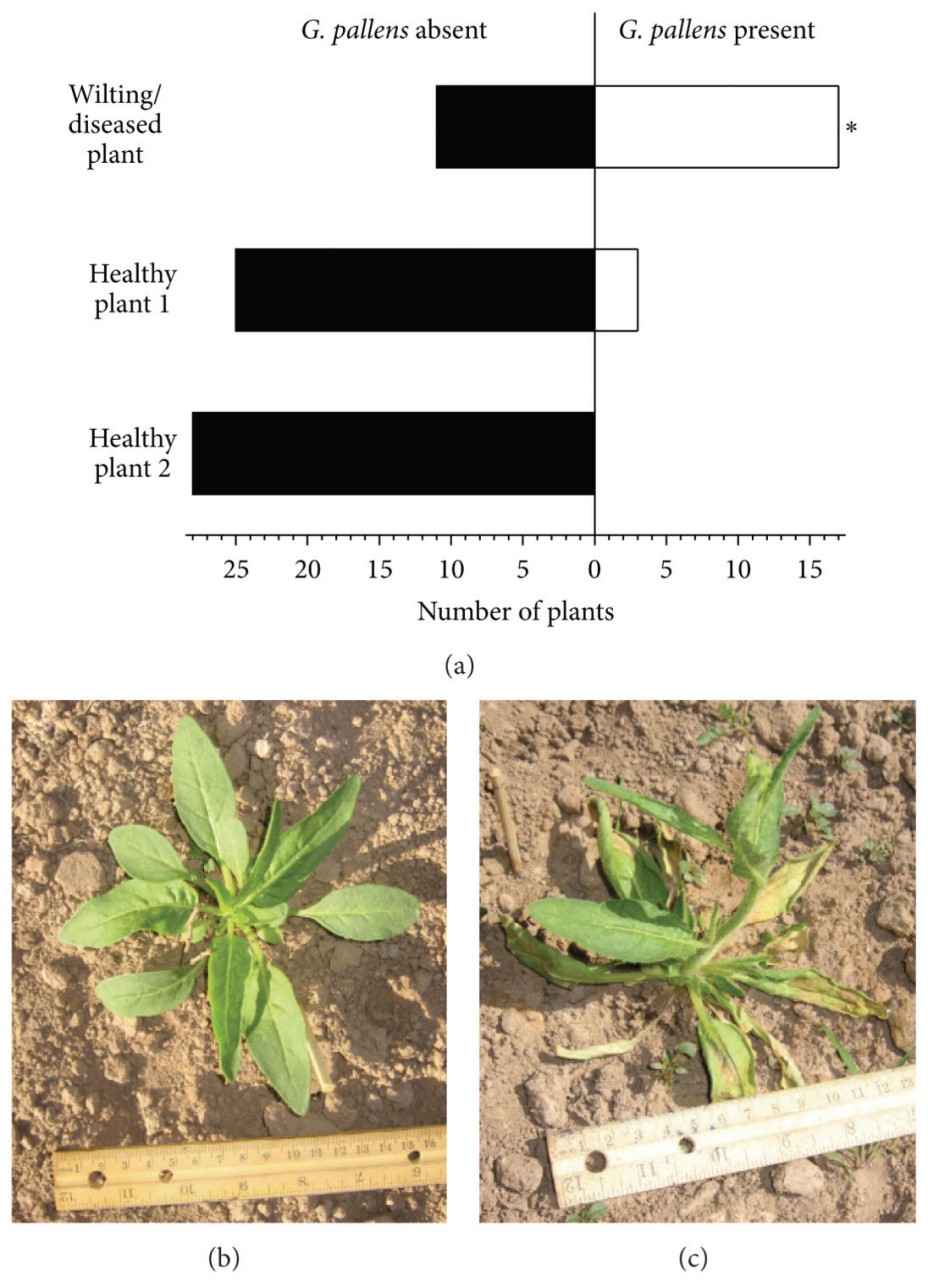
*G. pallens* is more likely to be found on wilting plants. The graph shows the number of wilting, diseased, and neighboring healthy plants found to harbor *G. pallens* individuals (a). Healthy plant 1 (b) was on average ca. 1.5 m, and healthy plant 2 was ca. 2-3 m away from the wilting plant (c). The asterisk indicates significant differences between the presence of *G. pallens* in both sets of healthy plants and wilting plants in a Fisher’s exact test, *P* < 0.0001.

In several years we observed *Geocoris* spp. individuals associated with diseased or damaged *N. attenuata* plants which began to wilt. In 2012, when a massive disease outbreak occurred which killed a huge number of plants, we investigated this phenomenon by counting the presence of *Geocoris* spp. on dying plants versus the two nearest healthy plants (Figure 7). On two days in May 2012 we searched for wilting plants and directly checked for the presence of *Geocoris* spp. and then checked the nearest neighboring healthy plant (approximately 1-2 m away) and the second nearest neighboring healthy plant (approximately 2-3 m away) for *Geocoris* spp. presence. Except for one plant, only single *G. pallens* individuals were found on plants.

### 2.6. G. pallens Survival on Leaf Tissue and Water versus Water Alone

**Figure 8:**
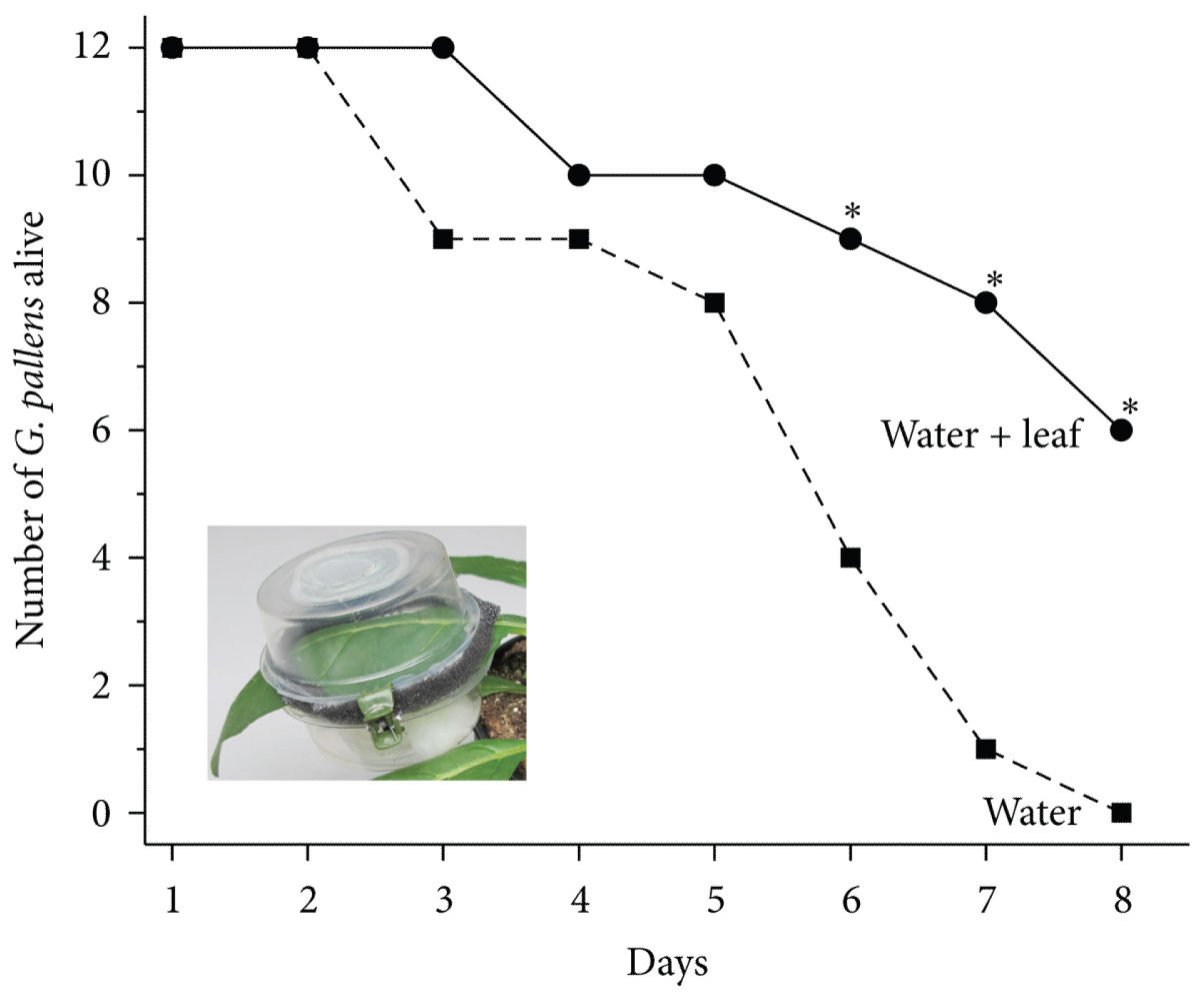
Use of *N. attenuata* leaf tissue as a food source by *G. pallens*. The graph shows the number of *G. pallens* individuals surviving in clip cages with either a cotton ball soaked with water (water) or water plus an *N. attenuata* leaf still attached to a living plant (water + leaf). Inset: clip cage on a leaf with a moist cotton ball in the lower right (water + leaf treatment); each half of the cage has a hole covered with netting to permit transpiration (only visible for top half). Asterisks indicate significant differences between treatments obtained by Fisher’s exact tests on indicated days, *P* < 0.05.

*Geocoris* spp. have been reported to feed on seeds and insects. In 2006, to test the potential of *G. pallens* to survive on leaf tissue, we conducted a feeding assay in which *G. pallens* adults collected in Lytle were offered either water from an underground spring, or spring water and an *N. attenuata* leaf (Figure 8). Each individual (*n* = 12 collected immediately prior to the start of the assay) was caged in a 50 mL food-quality plastic container (Huhtamaki; http://www.huhtamaki.com/) secured with miniature claw-style hair clips and padded on the rim with foam to avoid damaging plant leaves. These “clip cages” contained a cotton ball moistened with spring water or the moist cotton ball and part of an *N. attenuata* leaf. The leaf was still attached to a living plant and thus did not have to be replaced for the duration of the experiment. The plant did not harbor any insect herbivores. The cotton ball with water was exchanged every second day. Mortality was monitored once a day at noon.

### 2.7. Statistics

Fisher’s exact tests conducted using a spreadsheet (J. H. Macdonald, http://udel.edu/~mcdonald/statfishers.html) for Excel (Microsoft) [44] were used to compare counts of predated eggs, *M. sexta*-feeding *G. pallens*, plants harboring *G. pallens* individuals, and *G. pallens* individuals surviving on a water-only versus water and live leaf diet. When necessary, Bonferroni post hoc corrections were calculated using Excel to correct for multiple testing.

## 3. Results

### 3.1. Geocoris pallens and G. punctipes Populations at the Study Site

*G. pallens* and *G. punctipes* can be easily distinguished by differences in the coloration of their eggs and by the size and coloration of their nymph and adult stages (Figures 1 and 2). In 10 years of field research at Lytle Preserve and in the surrounding areas, we have almost exclusively found *G. pallens* associated with invasive *Erodium cicutarium* (L.) L’Hér. ex Aiton (Geraniales: Geraniaceae) plants and alfalfa (*Medicago sativa* Linnaeus, Fabales: Fabaceae) plantations in the early spring (mid-April to mid-May) and with *N. attenuata* plants in the late spring to summer (from the end of May). In contrast, we have observed *G. punctipes* primarily on *Cucurbita foetidissima* Kunth in Humb. (Cucurbitales: Cucurbitaceae) and *Datura wrightii* Regel (Solanales: Solanaceae) plants. We have not observed *Geocoris* spp. in areas where ants are abundant.

Our observations over several years indicate that the main food for *G. punctipes* on *C. foetidissima* is *Empoasca* spp., and on *D. wrightii*, *Lema trilineata* (Olivier) (Coleoptera: Chrysomelidae) eggs and *Manduca* spp. eggs and larvae. The main foods for *G. pallens* on *N. attenuata* appear to be *Epitrix* spp., *T. notatus*, *Manduca* spp. eggs and young larvae depending on their abundance, and, when plants are setting seed, *C. extensa*, the seed-feeding negro bug. On *N. attenuata*, *G. pallens* begin by eating primarily flea beetles (*Epitrix* spp.), which are usually the first herbivores on *N. attenuata*, and mirids (*T. notatus*) which arrive on *N. attenuata* as plants begin to elongate but will switch to eating *M. sexta* and *M. quinquemaculata* eggs and young larvae when *Manduca* spp. are abundant, usually after *N. attenuata* begins to flower and attract *Manduca* spp. as pollinators [23, 30]. We have also found *G. pallens* sheltering in open *N. attenuata* seed capsules overnight and eating ripe seed (M. C. Schuman and M. Stanton, observation). In 2008–2010 we observed that the number of *Manduca* spp. eggs preyed on *Geocoris* spp. increased in locations which received oviposition from native *Manduca* spp. moths (2008, M. C. Schuman and S. Allmann, observation; 2009, [30]; 2010, I. T. Baldwin and C. Diezel, observation). In 2011, we found that *Geocoris* spp. began to prey on *M. sexta* larvae within 24 h after plants were experimentally infested with larvae, in the absence of wild *Manduca* spp. oviposition [31].

We have observed that *Geocoris* spp. adults emerge from overwintering sites in March and April and lay eggs which hatch in May, giving rise to a second generation; the adults of this second generation overwinter to the following year. We have generated laboratory colonies of both *Geocoris* species from field collections. *G. punctipes* can be easily reared in captivity, and there are multiple other colonies of this species in captivity, primarily for use in biological control [4, 6, 9, 11–18, 20, 21, 33]. The *Geocoris* spp. in our colonies have similar developmental and survival times as reported in the literature (ca. 1 month for nymph development and 1–3 months survival as adults, see Section 1), and adults reproduce year-round.

### 3.2. Developmental Stages and Color Morphs of G. pallens

In the 2009 field collections of *G. pallens* we observed five nymphal stages (instars) (Figure 2) occasionally present as dark morphs (Figure 3(a)) but dominated by a light morph (Figure 3(b)). Adults from dark and light morphs were able to interbreed, and both morphs have since reoccurred in our colony in Jena, which is propagated from annual field collections in Lytle Preserve and the surrounding areas.

### 3.3. Feeding Behavior of G. pallens

A. Shillabeer with Merit Motion Pictures filmed one of our field-collected *G. pallens* feeding on *M. sexta* eggs and larvae in high-resolution macro focus (Figure 4). In these pictures, one can clearly see how the proboscis sheath is used to penetrate prey and then bends at three joints, permitting the flexible stylets to emerge and suck out the prey’s contents.

### 3.4. Geocoris spp. Predation Activity Varied Significantly within a Single N. attenuata Population

We found that *Geocoris* spp. predation of *M. sexta* eggs varied significantly for patches in a single wild *N. attenuata* population in 2007 (Figure 5, *n* = 146–167 eggs per site, pairwise Fisher’s exact tests followed by a Bonferroni correction for multiple testing: site 1 versus site 2, *P* = 0.0002; site 2 versus site 3, *P* < 0.0001; site 1 versus site 3, *P* = 0.0027). This difference was driven by total *Geocoris* spp. predation activity and not necessarily by the attractiveness of plants for *Geocoris* spp. in each site: between 91% and 100% of plants at each site had at least one egg predated. There were no significant differences among sites in the numbers of plants from which eggs were predated (*P* > 0.4).

### 3.5. G. pallens Generations Varied in Their Tendency to Eat M. sexta Eggs and Larvae, but Nymphs and Adults Did Not

We tested field collections of *G. pallens* from a native *N. attenuata* population in 2009 for their tendency to eat *M. sexta* eggs or larvae (Figure 6, Table 1). Between 61 and 81% of *G. pallens* collected and tested in the field in mid-May (15th or 16th) ate *M. sexta*. Collections from the same *N. attenuata* population were tested again in June, within 48 h after collection and transport to the lab in Jena. In a collection from June 1st, 32% of individuals ate *M. sexta*, and this increased slightly (but not significantly) to 37% in a collection from June 15th (*n* = 31–107 individuals per collection, pairwise Fisher’s exact tests followed by a Bonferroni correction for multiple testing: *P* < 0.0001 for the May 15th versus the June 1st collection, *P* = 0.0163 for the May 16th versus the June 1st collection, *P* < 0.0001 for the May 15th versus the June 15th collection, but *P* = 0.0699 [not significant] for the May 16th versus the June 15th collection). *G. pallens* individuals from collections made within the same month did not significantly differ in their tendency to eat *M. sexta* (*P* > 0.2). Mortality over the course of the 72 h assay was less than 15% and did not differ significantly among collections (pairwise Fisher’s exact tests followed by a Bonferroni correction for multiple testing, *P* > 0.06).

All collections comprised both adults and nymphs, and the May 15th collection had a particularly good representation of most nymphal stages and adults (Table 1). There was no significant difference among different developmental stages in their tendency to eat *M. sexta* eggs (Figure 6(c), pairwise Fisher’s exact tests followed by a Bonferroni correction for multiple testing, *P* = 1), although in the case of second-instar nymphs, which tended to eat fewer eggs, this was likely due to low replicate numbers.

### 3.6. G. pallens Individuals Associated with Wilting and Diseased Plants

When *N. attenuata* plants wilted in the field due to various stresses, for example, uprooting by wind, cattle damage, or disease, we often observed *Geocoris* spp. around the dying plants. When a fungal disease outbreak killed a large number of *N. attenuata* plants in 2012, we found *G. pallens* more frequently on wilting plants (Figure 7). *G. pallens* was present on 61% of the wilting plants, but only on 11% of the nearest healthy neighboring plants (1.5 m away), and no *Geocoris* spp. could be found on the second-nearest healthy plants (approximately 2-3 m away from wilting plants) in any of 28 replicates (Fisher’s exact test of numbers of healthy versus wilting plants harboring *G. pallens*, *P* < 0.0001).

### 3.7. G. pallens Can Use Leaf Tissue as a Food Source

*G. pallens* adults survived significantly longer if reared on *N. attenuata* leaves and a water-soaked cotton ball than only on the wet cotton ball. After six days without any insect prey, twice as many *G. pallens* individuals died if given only water than if given leaf material and water (*n* = 12 individuals per treatment, Fisher’s exact test, *P* = 0.0498; Figure 8). This effect lasted until the end of the experiment after eight days, when all *G. pallens* individuals living only on water had died. After seven days, only four individuals had died if they were allowed to feed on plant diet, while 11 individuals had died in the water-only group (*P* = 0.0047), and after eight days all animals reared only on water had died, while six individuals given *N. attenuata* leaves remained alive (*P* = 0.0069).

## 4. Discussion

We have observed aspects of the life history, host plant, and insect prey preferences of *G. pallens* and *G. punctipes* that co-occur with the native tobacco *N. attenuata* in the Great Basin Desert of southwestern Utah. For *G. pallens*, we have also captured images of feeding behavior with a high-resolution macro lens (courtesy of A. Shillabeer and Merit Motion Pictures), quantified variation in the predation of one insect prey species, *M. sexta*, in space and time, recorded increased occurrence around wilting or sick *N. attenuata* plants, and demonstrated the ability to survive on only water and vegetative plant tissue, which has not otherwise been demonstrated for any species of *Geocoris*. Furthermore, we describe how we have maintained laboratory colonies of both *Geocoris* species from field collections.

### 4.1. Geocoris pallens and G. punctipes May Have Overlapping Ranges but Separate Niches in Southwestern Utah

*G. pallens* and *G. punctipes* can be easily distinguished based on size and morphology at all life stages (Figures 1 and 2). We have found these species feeding on a partially overlapping diet of insect prey but almost always on different plant species: *G. pallens* is associated with *E. cicutarium* plants and *M. sativa* plantations in the early spring and with *N. attenuata* plants in the late spring and summer; this apparent host shift is likely due to the fact that *E. cicutarium*, a shallow-rooted ground cover, dries up by the end of May or beginning of June. We have observed *G. punctipes* primarily on *C. foetidissima* and *D. wrightii* plants. Both *Geocoris* spp. will feed on *Manduca sexta* and *M. quinquemaculata* eggs and young larvae, and both eat the same food in our colonies, but in nature their diets may overlap very little except for *Manduca* spp. and the mirid *T. notatus*.

Within the native populations of *G. pallens*, we have observed two color morphs (Figure 3). The light morph seems to be the more prevalent. It would be interesting to know whether the difference in pigmentation is genetically or environmentally based, because dark and light morphs co-occur in the same populations without any obvious differences in microclimate, a genetic basis seems likely. *G. pallens* nymphs and adults spend most of their time foraging on plants and moving between plants over the sandy ground or sheltering in the shade of plants. The dark morph may be better camouflaged in the shade, while the light morph would be better camouflaged on the sunlit sand. Potential behavioral differences associated with the color morphs, however, remain to be investigated. To our knowledge, such a strong color contrast has not been reported as a morphotype in any other species of *Geocoris*.

### 4.2. G. pallens Predation Activity Varied Significantly within an N. attenuata Population

We found that the number of *M. sexta* eggs predated by *G. pallens* (Figure 4) varied significantly for *N. attenuata* plants in different locations within a single population (Figure 5). This might have been due to local variation in *G. pallens* population density or differences in feeding behavior within a host plant population, perhaps dependent on local *Manduca* spp. oviposition events or differences in the abundance of other prey. We do not know how far *G. pallens* individuals travel in search of prey, but the assay sites we chose were ca. 50–100 m apart, and it is possible that *G. pallens* at the different sites represented local subpopulations with little exchange of individuals between them.

It is also possible that differences in *G. pallens* predation activity were due to differences in *N. attenuata* plant phenotypes. *N. attenuata* plants within a population vary greatly both in neutral genetic markers [45] and in their response to herbivore attack, particularly the volatiles they emit and their degree of induced defense upon herbivore feeding [46]; the variation within populations is as great as the variation between populations in these plant traits [28, 47]. *G. pallens* and *G. punctipes* respond to specific herbivore-induced volatiles of *N. attenuata* [22, 26], but it is not known how quickly or how well they learn to respond to the differing volatile profiles of plants in native populations. The phenomena of associative susceptibility and associative resistance, in which plant traits increase or decrease the herbivore loads of neighboring plants, are widespread in ecological communities [48], and associative susceptibility or resistance due to neighbor volatile emission may contribute to the site-by-site variation in *Geocoris* predation activity.

### 4.3. G. pallens Prey Choices May Be Learned Anew with Each Generation but Did Not Differ between Nymphs and Adults Tested Simultaneously

We tested field-collected *G. pallens* adults and nymphs from the same wild population over a season (Table 1) for their inclination to eat *M. sexta* eggs in no-choice assays (Figure 6). Based on known emergence and generation times for these insects, the May collections must have been from the first generation of eggs laid in 2009; we found that 61–81% of the individuals consumed *M. sexta* eggs in no-choice assays. From these collections, we can conclude that there is no significant difference between nymphs and adults in their propensity to eat *M. sexta* eggs, because the May 15th collection comprised 27% adults, whereas the May 16th collection comprised 71% adults (Table 1), and the two collections did not significantly differ in their tendency to eat *M. sexta* eggs (Figure 6(a)). Furthermore, different developmental stages within the May 15th collection also did not differ significantly in their tendency to eat *M. sexta* eggs (Figure 6(c)), although in the case of 2nd instar nymphs this was likely due to low replicate numbers. It should be noted that the composition of nymphs in collections may not accurately reflect the composition of the sampled *G. pallens* population: later nymphal stages and adults are probably overrepresented, because they are easier to see and catch.

The June 1st collection made two weeks later comprised 68% adults; in this collection, the nymphs were certainly the offspring of the May collections, and the adults may have been a mix of May-nymphs and May-offspring. This collection was transported to the lab in Jena for testing, and although they were allowed to adapt for 24–48 h, transport and laboratory conditions may have negatively affected feeding rates. Only 32% of these *G. pallens* fed on *M. sexta* eggs in the same no-choice assay. A final collection made two weeks later (June 15th) and also tested after transport to the laboratory comprised 95% adults, all of which were likely offspring of the May collections. In the June 15th collection, the number of egg feeders had increased slightly to 37%.

The May and June generations may have experienced separate *Manduca* spp. oviposition events which influenced their propensity to eat *M. sexta* eggs. There are typically two *Manduca* spp. oviposition peaks in the Lytle area and surroundings: one at the end of April to the first week of May (mainly on *D. wrightii*) and one in the middle of June (*D. wrightii* and *N. attenuata*). (*Manduca* spp. oviposition, however, occurs to a minor degree also between those two peaks.) Given that lepidopteran eggs are more nutritious for *Geocoris* spp. than aphids [5, 38] and likely other hemipteran prey such as *T. notatus*, it is interesting that *G. pallens* does not always readily eat *M. sexta* eggs but might need to learn to prey on them. *G. punctipes* seem to be strongly influenced by prey mobility rather than nutritional quality [38]; a preference for mobile prey might explain why *G. pallens* does not always seem to recognize *M. sexta* eggs as prey [23, 30]. Perhaps *G. pallens* must first learn to associate *Manduca* spp. eggs with feeding larvae and the associated herbivore-induced plant volatiles.

### 4.4. G. pallens May Scavenge from Dying Plants

We found *G. pallens* individuals to be significantly (5-fold) more abundant on dying, wilting *N. attenuata* plants than on nearby healthy plants. In fact, in a 2012 plant disease outbreak, *G. pallens* were not found on healthy plants unless they were next to wilting plants. This could be due to greater foraging success for *G. pallens* when hunting insects fleeing from dying plants, or it might be that dying plants are a better nutritional supplement to *G. pallens*’s insect diet, which may also be more nutritious when herbivores feed on dying plants. It has long been known that nutrients, including amino acids, are mobilized from water-stressed and senescing plant tissue, although some reports indicate that the lack of turgor pressure in wilting plants reduces sap flow so that phloem feeders may not be able to access these increased resources [49]. If *G. pallens* are feeding directly from cells or the apoplastic space, they may be able to harvest the products of cellular senescence and degradation from dying plants. A more speculative hypothesis would be that *G. pallens* itself transfers disease when feeding on plant tissue, as is known for herbivores (e.g., [42]). If *T. notatus* is more fecund on diseased plants, as a consequence of impaired host plant resistance, *Geocoris* could benefit from spreading disease and thus increasing the current population of its prey. However, herbivores likely spread plant disease more efficiently than omnivores such as *Geocoris* spp.

### 4.5. G. pallens Feeds on Seeds and Leaves

Although it has been reported that *Geocoris* spp. feed from vegetative plant tissue, all prior reports indicated that *Geocoris* individuals could not survive any better on vegetative tissue than on water alone [4]. Here, we show that mortality of *G. pallens* individuals offered water and living leaf tissue *on planta* is 50% over 8 d, but for individuals given only water is 100%. The discrepancy between our results and previous results could be due to the appropriateness of the plant tissue for the particular *Geocoris* spp.; differences in nutritional quality of cut leaves [4] versus leaves left on a plant; or even the increased importance of relative humidity provided by leaf cover in a desert environment, which is unlikely to be a factor in a laboratory. We have seen *G. pallens* individuals drinking from *N. attenuata* leaves in wild populations without leaving visible leaf damage (S. Allmann and M. C. Schuman, observation).

## 5. Conclusions

Wild populations of *G. pallens* change plant hosts and adapt to changes in host quality and herbivore prey abundance over their lifetimes. *G. punctipes*, though co-occurring with *G. pallens*, uses different host plant and herbivore resources than does *G. pallens* in southwestern Utah. *Geocoris* spp. are phenotypically plastic generalists which, though omnivorous, benefit plants by reducing their herbivore loads. These insects have become a model system to study the development of plant-herbivore-predator tritrophic interactions, and how predators learn plant cues, and have great promise as effective biological control agents for agriculture.

## Figures and Tables

**Table 1:** Distribution of nymphal and adult stages in *G. pallens* collections tested for their tendency to eat *M. sexta* eggs (Figure 6).

Stage	May 15th	May 16th	June 1st	June 15th
Nymphs				
1	—	—	—	—
2	8.1%	—	1.0%	—
3	16.1%	—	—	—
4	21.0%	—	9.2%	1.9%
5	27.4%	29.0%	21.4%	2.8%
Adults	27.4%	71.0%	68.4%	95.3%
*n*	**62**	**31**	**98**	**107**
